# Macrophage-derived extracellular vesicles as new players in chronic non-communicable diseases

**DOI:** 10.3389/fimmu.2024.1479330

**Published:** 2025-01-17

**Authors:** Fengjuan Lin, Huiyu Luo, Jiexian Wang, Qing Li, Longying Zha

**Affiliations:** ^1^ Department of Nutrition and Food Hygiene, Guangdong Provincial Key Laboratory of Tropical Disease Research, National Medical Products Administration (NMPA) Key Laboratory for Safety Evaluation of Cosmetics, School of Public Health, Southern Medical University, Guangzhou, Guangdong, China; ^2^ Department of Clinical Nutrition, Nanfang Hospital, Southern Medical University, Guangzhou, Guangdong, China

**Keywords:** macrophage-derived extracellular vesicles, exosomes, biological functions, drug delivery systems, chronic non-communicable diseases

## Abstract

Macrophages are innate immune cells present in all tissues and play an important role in almost all aspects of the biology of living organisms. Extracellular vesicles (EVs) are released by cells and transport their contents (micro RNAs, mRNA, proteins, and long noncoding RNAs) to nearby or distant cells for cell-to-cell communication. Numerous studies have shown that macrophage-derived extracellular vesicles (M-EVs) and their contents play an important role in a variety of diseases and show great potential as biomarkers, therapeutics, and drug delivery vehicles for diseases. This article reviews the biological functions and mechanisms of M-EVs and their contents in chronic non-communicable diseases such as cardiovascular diseases, metabolic diseases, cancer, inflammatory diseases and bone-related diseases. In addition, the potential application of M-EVs as drug delivery systems for various diseases have been summarized.

## 1 Introduction

### 1.1 Macrophages

Macrophages, including monocytes in the circulatory system and tissue-resident macrophages, are the most plastic immune cells in the hematopoietic system and are ubiquitous in all tissues. They play a crucial role in the entire spectrum of an organism’s biology, swiftly responding to various infections while also contributing to the development, homeostasis, initiation, maintenance, resolution of inflammation, tissue damage repair, and immunity (1–3). Tissue-resident macrophages are categorized based on their location and function, including microglia in the central nervous system, osteoclasts in bones, alveolar macrophages in the lungs and Kupffer cells in the liver (4, 5). Macrophages form different subtypes, such as M1 and M2, in different tissues depending on the environmental stimuli (6, 7), which represent the extremes of the polarization spectrum. Currently, an increasing number of studies have illuminated the existence of numerous intermediate polarization states within macrophages. M1 phenotypic macrophages are usually induced by interferon (IFN)-γ, lipopolysaccharide (LPS), granulocyte macrophage colony-stimulating factor, or tumor necrosis factor (TNF) alone or in combination *in vitro*. M1 polarization of macrophages alters the expression of a variety of cell surface markers such as cluster of differentiation (CD)80, CD86, CD36, inducible nitric oxide synthase (iNOS), and intercellular adhesion molecule 1. M1 phenotypic macrophages produce and secrete high levels of pro-inflammatory cytokines, which play an important role in the early stages of inflammation (6). In contrast, M2 phenotypic macrophages have anti-inflammatory effects. Depending on the activation stimulus, M2 macrophages can be further subdivided into different subtypes. M2 macrophages express a variety of cell surface markers, such as the mannose receptor (CD206), arginase-1, IL1R2, TLR-1, and TLR-8. Moreover, M2 phenotypic macrophages produce a variety of anti-inflammatory and pro-inflammatory cytokines (8). Functionally, M1 macrophages are able to eliminate infected cells (9), induce inflammatory responses, mediate tissue damage (10), impair tissue regeneration, and have antitumor activity (11, 12). M2 macrophages have a strong phagocytic ability, clear apoptotic cells, participate in the Th2 response and parasite clearance (13, 14), inhibit inflammation, and promote tissue repair, wound healing (10, 15, 16), angiogenesis, immunomodulation, and tumor formation and progression (17, 18). Macrophages play an important role in regulating the body’s diverse functions. Macrophage polarization is closely related to disease development, and polarization disorders may cause diseases. Macrophages with different phenotypes or responses to stimuli have been utilized for research purposes. However, macrophage-derived extracellular vesicles (EV) of different phenotypes exhibit behaviors similar to those of the parental cells, garnering widespread attention.

### 1.2 Macrophage-derived EVs

#### 1.2.1 Classification, characterization, and isolation of macrophage-derived EVs

EVs are membrane-bound particles important for intercellular communication and with potential applications in diseases and therapeutics (19). Macrophage-derived extracellular vesicles (M-EVs) have garnered attention due to their similarities to macrophages. Frequently utilized primary macrophages include murine bone marrow-derived macrophages (BMDM) (20), peritoneal-derived macrophages (PCM) (21, 22), adipose tissue-derived macrophages, or monocytes isolated from human peripheral blood (23). Passage cell lines commonly used are RAW264.7 (24), mouse mononuclear macrophages (25), and the THP-1 cell line (26, 27). According to the latest guidelines of the International Society of Extracellular Vesicles and current research, M-EVs are classified as exosomes, microvesicles, and apoptotic bodies (28, 29). Exosomes originate from endosomes via endocytosis and are released outside the cell with a 50–150 nm diameter. Microvesicles (MV) originate from the cytoplasmic membrane with a diameter of 100–1000 nm (30). Apoptotic bodies, with a diameter of about 100–5000 nm, are formed during programmed cell death (31–33).

M-EVs are characterized by their morphology, size, and marker proteins. Commonly used techniques to characterize the morphology of EVs include scanning electron microscopy, transmission electron microscopy, cryo-electron microscopy, atomic force microscopy, and dynamic light scattering (34). Nanoparticle tracking analysis and tunable resistance pulse detection can be used to measure the concentration and particle size distribution of M-EVs (34, 35). Marker protein identification is critical and is usually performed using western blotting and enzyme-linked immunosorbent assay for routine analysis. Recommended positive marker proteins for characterizing M-EVs include transmembrane (CD9, CD63, and CD81) and cytosolic proteins (tumor susceptibility gene 101 and programmed cell death 6-interacting protein). The International Society of Extracellular Vesicles has identified proteins such as heat shock protein, actin, tubulin, and glyceraldehyde 3-phosphate dehydrogenase that do not meet EV-specific components (28). Notably, the identification of M-EVs should be analyzed in combination with the above aspects rather than relying on a single aspect.

The isolation and purification of EVs is the first step in subsequent studies. EVs separation can be achieved using methods such as differential ultracentrifugation, density gradient centrifugation, immunoaffinity, coprecipitation (36), size exclusion chromatography (37), asymmetric flow field fractionation (38), and a newly expanded method of microfluidic filtration (35). Currently, differential ultracentrifugation is the most commonly used and considered the gold standard for EV isolation (39). Each method has its own advantages and limitations, which are detailed in related reviews (35). Notably, the pretreatment method differs for different samples, and it is necessary to select an appropriate EV separation method according to the experimental purpose and requirements.

#### 1.2.2 Biologically active substances carried in M-EVs

EVs transport various cargoes (RNA, proteins, lipids, and DNA) to nearby or distant recipient cells, influencing their function and intercellular communication. M-EVs contain numerous RNA, including microRNAs (miRNA), mRNA, circular RNAs (circRNA), long non-coding RNA (lncRNA), small interfering RNAs, small nucleolar RNAs, and transfer RNA (40). The mechanisms that control the specific loading of RNA species into exosomes rather complicated. Current research reports that miRNAs have sequence motifs, which control their localization into exosomes. The protein heterogeneous nuclear ribonucleoprotein A2B1 (hnRNPA2B1) specifically binds exosomal miRNAs through the recognition of these motifs and controls their loading into exosomes (41). The loading of miRNAs into exosomes can be modulated by mutagenesis of the identified motifs or changes in hnRNPA2B1 expression levels. Moreover, it has been reported that the YBX1 protein is intricately involved in the selective loading of specific microRNAs into exosomes (42).

Among EV inclusions, miRNAs are the most studied RNA in terms of function and biogenesis. They are a class of 17–24 nt short non-coding RNAs that bind to the 3 í-untranslated region of the target mRNA to regulate physiological and biochemical processes (43, 44). The levels of miRNA expression in EVs vary under various physiological and pathological conditions. For example, the expression of miR-142-3p in plasma exosomes of patients with idiopathic pulmonary fibrosis is significantly higher than in normal individuals (45). The miRNAs in M-EVs play a role in the progression of many diseases and have the potential to serve as diagnostic markers (46, 47). The mRNA delivered by M-EVs can be translated into recipient cells, serving as a protein source for regulation in recipient cells (48). As key competitive endogenous RNAs, lncRNAs act as sponges for miRNAs to negatively regulate miRNAs and thus exert their function (49, 50). Studies have confirmed that lncRNAs in M-EVs exert physiological effects in various diseases such as tumors, inflammatory diseases, and myocardial infarction (51–53). In M-EVs, circRNA also acts as an endogenous RNA that competitively binds to miRNA to affect the expression level of the target mRNA, thereby exerting physiological effects (54–56). M-EVs also influence disease progression by delivering functional proteins (57). Many studies have reported how EVs interact with recipient cells and deliver functional cargo (e.g., RNA, protein) via multiple EV uptake pathways including direct membrane fusion, clathrin-mediated endocytosis, macropinocytosis, caveolin-mediated uptake, phagocytosis, lipid raft-mediated internalization, and direct transfer via tunneling nanotubes (58, 59).

## 2 Biological role of M-EVs in chronic non-communication diseases

### 2.1 Cardiovascular diseases

Atherosclerosis (AS) and myocardial infarction (MI) are prevalent cardiovascular conditions. AS is a chronic inflammatory disease of the large and medium-sized arteries with many known risk factors, including hypercholesterolemia, hypertension, diabetes, and smoking (60). AS is a chronic inflammatory process caused by the interaction of atherogenic lipoproteins with vascular wall cells (including endothelial cells, smooth muscle cells, and monocytes/macrophages) (61). Endothelial pro-inflammatory activation and endothelial cell dysfunction are important factors in the pathophysiology of AS (62). Recent studies have shown that M-EVs and their contents are involved in the pathogenesis of AS through a variety of pathways such as the regulation of glycolytic activity, endothelial vascular injury, inflammation, oxidative stress, hematopoiesis, neutrophil extracellular traps (NET), and macrophage infiltration (**Table 1**).

**Table 1:** Summary of the biological functions of M-EVs in cardiovascular diseases.

EV source	Effective molecules	Model system	Mechanism	Biological function	Reference
M2-BMDM-Exo	miRNA-99a/146b/378a	ApoE^−/−^ mouse model	Reduces TNF-α and NF-κB signaling, hematopoiesis and myeloid inflammation	Relieves AS	(80)
HG-BMDM-Exo	–	ApoE^−/−^mouse model/HSC	Increases glycolytic activity, hematopoiesis, circulating myeloid cell numbers, and atherosclerotic lesions	Promotes the progression of AS	(79)
RAW264.7-Exo	miR-16-5p	AS mouse model	Targets SMAD7	Facilitates inflammatory response and oxidative stress, aggravates AS progression	(73)
M2-THP-1-Exo	miR-221-3p	HUVECs/HUVEC cell injury model	–	Promotes HUVEC cell proliferation, inhibits HUVEC cell inflammation and apoptosis	(71)
RAW264.7-EVs	VEGF/Wnt3a/miR-210/miR-126/miR-130a	SVEC4-10EHR1/Matr-igel plug mouse model	Induces proliferation, migration, and tube formation in endothelial cells	Promotes angiogenesis	(72)
oxLDL-THP-1-Exo	–	HUVECs	–	Attenuates the growth and tube formation of endothelial cells	(64)
PAM-THP-1-EVs	miR-185-3p	AS rat models	Targets Smad7	Aggravates the development of AS in ApoE−/−mice	(69)
THP-1-Exo	–	MOVAS/HASMC	Induces VSMC TGFBR1 upregulation by suppressing let-7b-5p	Promotes vascular calcification occurrence	(75)
PMA/ox-LDL-THP-1-EVs	miR-199a-5p	HAEC/ApoE^−/−^ mice	Targets the SMARCA4/PODXL/NF-κB axis	Expedites endothelial cell pyroptosis and further accelerates AS	(65)
PMA-U937-Exo	miR‐4532	HUVECs	Targets SP1, activating NF‐κB P65	Promotes AS	(63)
oxLDL-THP-1-EVs	miR-146a	Naïve macrophages	Targets IGF2BP1 and HuR	Inhibits macrophage migration, accelerates the development of atherosclerosis	(66)
oxLDL- J774a.1 -EVs	integrins β1 and α5	VSMCs	Activates the ERK and Akt pathways	Promotes VSMC migration and adhesion	(77)
IL4-THP-1-Exo	miR-21/99a/146b/378a	3T3-L1/BMDM/Obese mice	Modulates miRNA levels, regulating energy metabolism and inflammatory signaling	Alleviates cardiometabolic inflammation in diet-induced hyperlipidemic obesity and insulin-resistant mice	(97)
PMA-THP-1/RAW264.7-EVs	miR-503-5p	HCAECs/HCASMCs	Downregulates Smad7, smurf1, and smurf2 and elevates TGFβ1	Aggravates AS	(70)
LPS-RAW264.7-EVs	–	MOVAS-1	Induces pro-inflammatory and pro-oxidative responses	Aggravates the VC process	(74)
oxLDL-THP-1-Exo	miRNA-146a	Neutrophils/Mouse AS model	Inhibits SOD2 expression, increases ROS production in neutrophils, induces NET formation	Deteriorates AS progression	(67)
Nicotine-RAW264.7-Exo	miR-21-3p	VMSCs/AS model	Targets PTEN	Promotes VSMC migration and proliferation, accelerates the development of AS	(78)
M1-BMDM-EVs	lncRNA MALAT1	Myocardial microvascular endothelial cells (MMECs)/MI mouse models	Activates the MEK/ERK pathway by carrying lncRNA MALAT1 and competitively binding to miR-25-3p	Inhibits angiogenesis and myocardial regeneration after MI	(51)
M2-PCMExo	miR-148a	Neonatal rat cardiomyocytes/MI/R rat model	Downregulates TXNIP, inactivates the TLR4/NF-κB/NLRP3 inflammasome signaling pathway	Mitigates MI/R injury	(85)
M2-BMDM-sEV	miR-181b-5p	HCAEC/Ischemia‐reperfusion (I/R) models	Polarizes cardiac‐resident CCR2 macrophages to an M2‐like phenotype	Improves myocardial tissue repair	(86)
M1-THP-1/RAW264.7-Exo	miR-155	MI model	Downregulates the RAC1–PAK1/2 and Sirt1/AMPKα–eNOS pathways	Declines the migration, proliferation, and tube formation of Ecs and depresses angiogenesis	(82)
M2-Exo	miR-1271-5p	AC16/AMI mouse model	Downregulates SOX6	Suppresses apoptosis of cardiomyocytes and promotes cardiac repair in AMI	(239)
M1-BMDM-Exo	miR-155	Cardiac fibroblasts/Myocardial infarction model	Downregulates Sos1 protein expression	Suppresses fibroblast proliferation and increases fibroblast inflammation	(83)
M2-BMDM-sEV	circUbe3a	CF/AMI animal model	Targets the miR-138-5p/RhoC axis	Promotes the proliferation, migration, and phenotypic transformation of CFs	(88)

Oxidative low-density lipoprotein (oxLDL)-stimulated macrophage-derived extracellular vesicles (oxLDL-M-EVs) accelerate AS progression by mediating endothelial cell injury, macrophage accumulation, oxidative stress, and NETs (63–67). Liu et al. employed oxLDL to induce macrophage foam cells and release exosomes with high levels of miR-4532. This miRNA is delivered to endothelial cells via exosomes, targeting specific protein 1 to activate NF-κB p65 signaling, promoting vascular cell adhesion molecule-1 expression, and inducing endothelial dysfunction (63). Huang et al. showed that oxLDL-M-EVs attenuated endothelial cell growth and tube formation (64). The expression of miR-199a-5p decreases in oxLDL-M-EVs, and its downregulation promotes endothelial cell pyroptosis by enhancing SWI/SNF related, matrix associated, actin dependent regulator of chromatin, subfamily a, member 4 expression, further accelerating AS progression (65). The oxLDL-M-EVs reduce macrophage migration and promote the capture of macrophages in blood vessel walls; miR-146a may inhibit macrophage migration by targeting IGF2BP1 and human antigen R (66). Another study revealed significant upregulation of miRNA-146a in the serum-derived exosomes of patients with AS and oxLDL-M-EVs. MiR-146a could also induce oxidative stress to promote the formation of NETs by inhibiting superoxide dismutase 2 expression, further exacerbating the progression of AS (67). This suggests that the same miRNA can perform the same functions in multiple ways.

The inhibition of endothelial cell migration and proliferation associated with AS development. M-EVs inhibit endothelial cell migration by mediating the transport of integrin β1, thereby inhibiting the collagen-induced mitogen-activated protein kinase/extracellular signal-regulated kinase signaling pathway (68). MiR-185-3p and miR-503-5p are significantly upregulated in M-EVs and also inhibit endothelial cell proliferation and migration (69, 70). M2-EVs promote endothelial cell proliferation, and miR-221-3p-rich M2-EVs inhibit apoptosis and inflammation of HUVECs, and promote their proliferation; the mechanism may be that miR-221-3p targets inhibition of Grb10 expression, but further experiments are needed to confirm this (71). Another study showed that M-EVs promoted endothelial cell proliferation, migration, and tube formation *in vitro* (72).

Oxidative stress is closely related to inflammation, and M-EVs promote AS development by inducing oxidative stress and inflammation (67, 69, 73, 74). Macrophage-derived exosomes (M-Exo) miR-16-5p promotes the inflammatory response and oxidative stress in ApoE-/- mice, exacerbates pathological changes, and increases apoptosis by targeting Smad7 (73). Similarly, M1-EVs miR-185-3p accelerates AS progression by downregulating Smad7, aggravating pathology and endothelial cell adhesion in ApoE-/- mice, and enhancing lipid levels, oxidative stress, and inflammation (69). Yaker et al. reported that LPS stimulation of macrophage-derived extracellular vesicles (LPS-M1-EVs) could induce inflammation and oxidative stress in vascular smooth muscle cells (VSMCs), thus exacerbating vascular calcification (VC) (74). VC is a vascular complication commonly observed in patients with diseases such as AS and chronic kidney disease. High phosphate stimulation of M-EVs promotes VC in VSMCs (75). New et al. showed that macrophage-derived stromal vesicles enriched with S100A9 and annexin V contribute to atherosclerotic intimal microcalcification (76).

Atherosclerotic plaque formation is associated with VSMC migration and proliferation. Plasma EVs from patients with AS promote adhesion and migration of VSMCs. Macrophage foam cells release more EVs than normal macrophages, transporting integrins to VSMCs and activating the extracellular signal-regulated kinase (ERK) and Akt pathways, thus enhancing VSMC migration and adhesion (77). This indicates that macrophages in atherosclerotic lesions influence VSMCs via EVs, prompting their migration to and adhesion within the intima. Moreover, exosomes from nicotine-treated macrophages increased VSMC migration and proliferation by delivering miR-21-3p and targeting phosphatase and tensin homolog (PTEN), thereby accelerating AS (78).

Bouchareychas et al. investigated the effects of high glucose (HG)-BMDM-EVs on AS and found that they induced macrophage and myeloid progenitor cell proliferation, and regulated energy metabolism *in vitro* by increasing glycolytic activity. Infusion of HG-BMDM-EVs into ApoE-/- mice was found to enhance AS by increasing hematopoiesis, bone marrow cell supply, lesion macrophage content, and apoptotic cell number (79). IL-4-polarized bone marrow macrophage-derived exosomes (IL-4-BMDM-Exo) play a protective role by containing anti-inflammatory miRNAs (miRNA-99a/146b/378a), which inhibit inflammation by targeting NF-κB and TNF-α signaling. Infusion of IL-4-BMDM-Exos into ApoE-/- mice reduced transitional hematopoiesis in the bone marrow, thereby reducing the number of circulating myeloid cells and macrophages in aortic root lesions, resulting in reduced area of necrotic lesions in AS (80). This suggests that M-EVs are a promising treatment modality for AS.

Several studies have shown that M-EVs play an important role in mediating the immunomodulatory processes of MI and post-MI remodeling. In the MI microenvironment, mesenchymal cells effectively treat acute myocardial infarction (AMI) by influencing cell differentiation, migration, angiogenesis, and repair. Studies have shown that M1-Exo induced by hypoxia and serum deprivation (H/SD-M1-Exo) induce apoptosis in bone marrow mesenchymal stem cells (BMSCs) by delivering miR-222 (81). This affects the efficacy of BMSCs in AMI treatment. In a mouse model of MI, M1-BMDM-EVs increased CDC42 expression by carrying the lncRNA MALAT1 and competitively binding to miR-25-3p to activate the MEK/ERK pathway, thereby inhibiting angiogenesis and myocardial regeneration after MI (51). In the same model, M1-Exo miR-155 was transferred into endothelial cells (ECs), and miR-155 reduced the angiogenesis of ECs and exacerbated myocardial injury by simultaneously targeting the Rac family of small GTPases 1 (RAC1), p21 (RAC1)-activated kinase 2 (PAK2), sirtuin1 (Sirt1), and protein kinase AMP-activated catalytic subunit α2 (82). M1-Exo miR-155 can be transported not only to Ecs but also to cardiac fibroblasts, inhibiting fibroblast proliferation and promoting fibroblast inflammation by downregulating Sos1 expression, thereby impairing cardiac repair after MI (83). Interestingly, miR-155, an exosome derived from the adipose tissue macrophages in obese mice, was previously described to promotes obesity-induced insulin resistance. In distinct cell types, a single miRNA may bind to varying target genes, leading to contrasting biological effects. This phenomenon underscores the complexity and diversity of miRNA functions, which depend not only on miRNA expression levels but also on cell-specific regulation and interactions. Environmental conditions such as intracellular signaling pathway activity and epigenetic modification may also affect the mode of action of miRNAs (84). It may lead to difficulties in further clinical research. Studies have demonstrated that M2-EVs contribute to ventricular remodeling following MI. In a rat model of myocardial ischemia/reperfusion injury (MI/R), M2-Exos miR-148a are transported to cardiomyocytes and attenuates MI/R by downregulating thioredoxin-interacting protein (TXNIP) and inhibiting the TLR4/NF-κB/NLRP3 inflammasome signaling pathway (85). Another study has suggested that M2-EVs exert protective effects against ischemia-reperfusion cardiac injury. The main mechanism is that M2-EVs polarize heart-resident CC chemokine receptor 2 (CCR2) macrophages to the M2 phenotype, inhibit excessive glucose uptake and mtROS production by CCR2 macrophages, promote myocardial neovascularization, and facilitate cardiac repair after AMI, in which miR-181b-5p plays a key role, but further studies are needed to confirm this (86). In a mouse AMI model, M2-Exos carrying miR-1271-5p alleviated hypoxia-induced cardiomyocyte apoptosis and promoted cardiac repair of AMI by downregulating SOX6 expression (87). Wang et al. studied the effects of M2-EVs on cardiac fibroblasts and found that circUbe3a was overexpressed in M2-EVs, promoting the proliferation, migration, and phenotypic transformation of cardiac fibroblasts by directly targeting the miR-138-5p/RhoC axis, thus exacerbating myocardial fibrosis after MI (88). In summary, M-EV-mediated cell-to-cell communication plays an important role in the progression of AS and MI.

### 2.2 Metabolic diseases

Obesity and type 2 diabetes mellitus (T2D) are common metabolic diseases, and a growing body of research suggests that M-EVs play an important role in these metabolic diseases by mediating inflammation and regulating insulin sensitivity, lipid metabolism, glucose uptake, and mitochondrial activity (**Table 2**).

**Table 2:** Summary of the biological functions of M-EVs in metabolic diseases.

EV source	Effective molecules	Model system	Mechanism	Biological function	Reference
LPS-THP-1-Exo	–	Human omental adipocytes	–	Induces changes in the expression of genes implicated in pathways related to inflammation, carbohydrate catabolism, and cell activation regulation	(102)
ATM-Exo	miR-29a	3T3-L1/L6 myocytes/Primary hepatocytes/Obese and lean mouse models	Targets PPAR-δ	Promotes obesity-induced insulin resistance	(91)
ATM-Exo	miR-210-5p	Primary hepatocyte/3T3-L1/L6/CUG-SGA rats	Targets SIDT2	Promotes insulin resistance in CUG-SGA rats	(92)
M2-BMDM-Exo	miR-690	3T3-L1/L6/Primary hepatocytes/Obese mice	Targets Nadk	Promote insulin sensitivity	(94)
ATM-Exo	miR-155	3T3-L1/L6/Primary hepatocytes	Targets PPARγ	Modulate *in vivo* and *in vitro* insulin sensitivity	(90)
M1-THP-1-MVs	–	Human primary mature adipocytes/Primary differentiated adipocytes	Activates the NF-κB pathway	Reduces insulin signal transduction, decreases insulin-stimulated glucose uptake	(98)
HFD-ATM-Exo	miR-140-5p	H9c2 myocardial cells/Obese mice	Targets SLC7A11, inhibits GSH synthesis	Alleviate ferroptosis and cardiac injury	(103)
HG-RAW264.7-EVs	miR-21-5p	MPC5 podocytes	Targets inhibition of A20, elevates the inflammasome NLRP3, caspases-1 and IL-1β	Causes podocyte injury in diabetic nephropathy	(104)
M2-RAW264.7-Exo	miR-25-3p	Mouse podocyte cells	Inhibits DUSP1 expression, activates autophagy in podocytes	Improves HG-induced podocyte injury	(108)
M1- BMM-Exo	miR-143-5p	Hep1-6	Inhibits MKP5 expression	Induces insulin resistance in hepatocytes	(93)
RAW264.7-Exo	–	HUVECs/Diabetic rat wound models	Inhibits secretion of pro-inflammatory enzymes and cytokines	Improves angiogenesis and re-epithelialization in diabetic wounds	(111)
M1-BMDM-Exo	miR-212-5p	MIN6	Targets SIRT2 and inhibits the Akt/GSK-3β/β-catenin pathways	Impairs beta cell insulin secretion	(101)
HG-RAW264.7-Exo	MALAT1	RAW264.7/Carotid artery balloon injury model in diabetic rats	Inhibits the expression of MiR-150-5p and upregulates the expression of resistin	Induces insulin resistance, promotes carotid artery plaque formation induced by balloon injury	(95)
HG-RAW264.7-Exo	miR-210	3T3-L1/Obese diabetic model	Targets NDUFA4 gene expression	Promotes mouse obese diabetes pathogenesis	(96)
HG-THP-1-sEV	miR-503	HUVEC/Mouse skin wound model	Inhibits IGF1R expression	Leads to HUVEC dysfunctions and hinders diabetic wound healing	(109)
M2-BMDM-Exo	–	BMSCs/Femur fracture model	Activates the PI3K/AKT pathway, induces macrophage polarization	Accelerates fracture healing in diabetic mice	(113)
M2-RAW264.7-Exo	miR-93-5p	Podocytes	Regulates the miR-93-5p/TLR4 axis	Attenuates LPS-induced podocyte apoptosis	(240)
ATM-Exo	miR-222-3p	RAW264.7/Wound healing model/db/db mice	Reduces Bim expression, regulates macrophage polarization	Promotes diabetic wound healing	(112)
dDMDM-Exo	miR-144-5p	BMSCs/T2DM rat model/Transverse femur shaft fracture rat model	Targets smad1	Suppresses bone repair and regeneration *in vitro* and *in vivo*	(110)
HG-RAW264.7-Exo	miR-7002-5p	mTEC	Targets Atg9b, inhibits autophagy	Induces renal tubular dysfunction and inflammation	(107)
HG-RAW264.7-Exo	TGFβ1 mRNA	Mesangial cell line (SV40 MES 13)/C57BL/6 mice	Activates TGFβ1/Smad3 pathway	Promotes mesangial expansion and renal fibrosis	(106)
LPS+PA-BMDM-Exo	miR-27-3p	3T3-L1/L6 myocytes/Primary hepatocytes/HEK293 cells	Targets Miro1	Promotes mitophagy deficiency-mediated inflammation and insulin resistance	(99)
HG-RAW264.7-Exo	–	SV40 MES 13)/DN model/C57BL/6 mice	Promotes NLRP3 inflammasome activation and autophagy deficiency	Accelerates kidney injury in mice with diabetic nephropathy	(105)

Obesity-induced insulin resistance is a key factor in the pathogenesis of T2D (89). M-Exos from the adipose tissue of obese mice containing miR-155 promoted obesity-induced insulin resistance. In contrast, M-EVs from the adipose tissue of lean mice attenuated obesity-induced insulin resistance (90). MiRNAs encapsulated within M-EVs derived from obese mice constitute a sophisticated paracrine and endocrine signaling network, capable of transmitting molecular cues to insulin-responsive tissues. This system intricately modulates glucose tolerance by directly repressing specific target genes, thereby influencing metabolic regulation. MiR-29a and miR-210-5p are overexpressed in adipose tissue-derived exosomes and are delivered to adipocytes, muscle cells, and hepatocytes, mediating insulin signaling by targeting peroxisome proliferator-activated receptor-δ and SID1 transmembrane family member 2, respectively, thereby impairing glucose cellular uptake and inducing insulin resistance *in vitro* and *in vivo* (91, 92). However, there is a paucity of research on the role of specific miRNAs in lean adipose tissue-derived M-EVs in enhancing insulin sensitivity, warranting further exploration. Bone marrow macrophage-derived exosomes regulate insulin sensitivity. MiR-143-5p is significantly upregulated in M1-polarized bone marrow macrophage-derived exosomes (M1-BMDM-Exo) in high-fat-fed mice, promoting hepatocyte insulin resistance by targeting mitogen-activated protein kinase phosphatase-5 (MKP5), thereby reducing AKT and glycogen synthase kinase activation and glycogen production in hepatocytes (93). A separate study demonstrated that M2-polarized bone marrow macrophage-derived exosomes (M2-BMDM-Exo) improved insulin sensitivity. Administration of M2-BMDM-Exo to obese mice improved glucose tolerance and insulin sensitivity. MiR-690 is highly expressed in M2-BMDM-Exo and enhances insulin sensitivity *in vivo* and *in vitro* by targeting NAD⁺ kinase, suggesting its potential as a novel therapeutic insulin sensitizer (94). Metastasis-associated lung adenocarcinoma transcript 1 (MALAT1) and miR-210 are significantly upregulated in hyperglucose-induced macrophage-derived exosomes (HG-M-Exo), contributing to insulin resistance (95, 96). MALAT1, a conserved long noncoding RNA (lncRNA), plays an important role in diabetes-related complications. HG-M-Exo MALAT1 induces insulin resistance by inhibiting miR-150-5p expression, thereby upregulating resistin expression (95). MiR-210 directly binds to the mRNA sequence of NADH dehydrogenase ubiquinone 1α subcomplex 4 (NDUFA4), inhibiting its expression in 3T3-L1 adipocytes, impairing glucose uptake and mitochondrial complex IV activity, and promoting the pathogenesis of obesity and diabetes in mice (96). However, Phu et al. found that IL-4-polarized human macrophage-derived exosomes (IL-4-THP-1-Exo) promote browning during the differentiation of 3T3-L1 preadipocytes into adipocytes by inducing adipogenesis and improving mitochondrial activity, glucose metabolism, and insulin resistance in obese mice (97). M1-EVs have been shown to induce insulin resistance through inflammation-related signaling pathways. For example, M1-EVs contribute to obesity-induced insulin resistance by activating the NF-κB signaling pathway, reducing insulin signaling, and decreasing glucose uptake in human adipocytes (98). M1-Exo miR-27-3p contributes to the development of T2D in mice by targeting mitochondrial Rho GTPase 1 to promote mitophagy damage-mediated inflammation, macrophage activation of the inflammatory M1-like phenotype *in vivo*, and insulin resistance (99). Chronic low-grade inflammation is the main cause of obesity-induced insulin resistance (100), contributing to islet β cell dysfunction. MiR-212-5p is significantly up-regulated in M1-BMDM-Exo and islet-resident M-Exos in high-fat-fed mice, impairing insulin secretion in beta cells by targeting the sirtuin2 gene and inhibiting Akt/GSK-3β/β-catenin signaling pathway (101). Additionally, LPS-activated M-Exos upregulated inflammation-and carbohydrate catabolism-related genes in adipocytes (102). M-EVs affect not only the progression of obesity and diabetes but also the development of associated complications. Zhao et al. found that exosomes derived from the adipose tissue macrophages of obese mice induced ferroptosis by delivering miR-140-5p and targeting solute carrier family 7 member 11 (SLC7A11) to inhibit glutathione synthesis, contributing to obesity-induced myocardial dysfunction (103).

Diabetic nephropathy (DN) is one of the most common microvascular complications of diabetes. Inflammasome activation-mediated podocyte damage plays an important role in DN. Ding et al. analyzed the effect of HG-M-Exo on mouse podocyte cell lines and found that miR-21-5p increased the expression of inflammasome NLR family pyrin domain containing 3 (NLRP3), caspase-1, and IL-1β in podocytes by inhibiting anti-inflammatory factor A20, thus causing podocyte damage in diabetic nephropathy (104). Similarly, HG-M-Exos promote inflammatory responses and accelerate kidney injury in DN mice by promoting NLRP3 inflammasome activation and autophagy deficiency in mesangial cells (105). Mesangial cell dysfunction is also associated with DN pathophysiology. TGFβ1 mRNA is highly expressed in HG-M-Exo and is transferred to mesangial cells to activate the TGFβ1/Mothers against decapentaplegic homolog (Smad)3 pathway, leading to mesangial cell activation and proliferation and secretion of extracellular matrix and inflammatory cytokines, promoting mesangial expansion and renal fibrosis (106). In addition, HG-M-Exo miR-7002-5p reduces renal tubular epithelial autophagy by targeting autophagy related 9B, thereby inducing tubular dysfunction and inflammation and aggravating DN (107). M2-Exos attenuate HG-induced podocyte damage and miR-25-3p in M2-Exo activates autophagy in podocytes by inhibiting dual specificity phosphatase 1 (DUSP1) expression, thereby ameliorating HG-induced podocyte injury (108).

A prevalent complication of diabetes is impaired healing. Studies have shown that M-EVs aid in the healing of diabetic wounds and fractures. High-glucose-induced M1-polarized macrophage-derived exosomes (HG-M1-Exo) transport miR-503 into HUVECs, which targets insulin-like growth factor (IGF)1R expression, which results in HUVEC dysfunction and impedes wound healing in diabetic patients (109). Bone marrow M-Exos miR-144-5p in diabetic rats inhibits the osteogenic differentiation of BMSCs osteogenic differentiation by inhibition of Smad1 expression *in vivo* and *in vitro*, thereby impairing the fracture healing process (110). M-EVs also contribute to the restoration of diabetic wounds and fracture healing by significantly reducing pro-inflammatory cytokine secretion, inducing endothelial cell proliferation and migration, accelerating angiogenesis, and promoting wound repair (111). Xia et al. found that administering lean mouse adipose tissue M-Exos to diabetes-prone db/db mice enhances wound healing. Subsequent studies revealed that miR-222-3p expression is significantly upregulated in exosomes, which regulates macrophage polarization and promotes rapid healing of diabetic wounds by targeting the inhibition of Bcl-2-like protein 11 expression (112). Similarly, M-EVs have been shown to expedite diabetic fracture healing by modulating macrophage polarization. M2-Exos induce macrophage M2 polarization by activating the phosphoinositide 3-kinase (PI3K)/(protein kinase B) AKT pathway, thereby reducing the proportion of M1 macrophages, significantly regulating the bone immune microenvironment, and accelerating diabetic fracture healing (113).

In summary, EVs and their endocannabinoids derived from different phenotypes or sources of stimuli play important roles in the development of metabolic diseases and complications through multiple pathways.

### 2.3 Role of M-EVs in cancer

Tumorigenesis depends, in part, on M-EV interactions with components of the tumor microenvironment (TME), and tumor-associated macrophages (TAM) are one of the most abundant immune cells in the TME and play an important role in tumorigenesis and progression (114). TAMs are divided into M1-like and M2-like phenotypes and have a special transition period from M1-like to M2-like phenotypes throughout tumor progression (115). M1 phenotypic TAMs exert pro-inflammatory and antitumor activity by secreting a variety of inflammatory cytokines (e.g. IL-6, TNF-α, IL-12, and ROS) and are associated with a favorable prognosis for cancer (116, 117). M2 phenotypic TAMs exert tumor promoting activity by inhibiting immune and inflammatory responses in cancer (117). Recent studies have shown that TAM-derived extracellular vesicles (TAM-EVs) play an important role in the TME by mediating tumor cell proliferation and invasion, angiogenesis, chronic inflammation, immunosuppression, and drug resistance (**Table 3**). The effects of EVs derived from TAMs with different phenotypic TAMs (M1/M2-EVs) on various cancers are reviewed below.

**Table 3:** Summary of the biological functions of M-EVs in cancer.

EV source	Effective molecules	Model system	Mechanism	Biological function	Reference
THP-1/PBMC-Exo	miR-142/miR-223	HuH7/HepG2/P815 cell lines	Decreases expression of STMN-1, IGF-1R	Inhibits HCC proliferation	(23)
M2-THP-1-Exo	miR-27a-3p	HCC cell lines (Huh7, 97H, HepG2, LM3, SMMC-7721)/Male BALB/c nude mice	Downregulates TXNIP	Induces HCC progression	(119)
PMA-THP-1-Exo	miR-92a-2-5p	SK-HEP-1/Hep G2/Hepa 1-6/HEK 293 T	Targets AR, altering the PHLPP/p-AKT/β-catenin signaling	Increases liver cancer cells invasion	(123)
M2-EVs	miR-21-5p	Hep1-6 cells/Primary HCC model	Targets YOD1 and activates the YAP/β-catenin pathway	Promotes CD8 T cell exhaustion in HCC	(121)
M2-THP-1-Exo	miR-660-5p	HepG2/Bel-7402/Xenograft nude mice model	Downregulates KLF3 expression	Augments hepatocellular carcinoma development	(120)
RBPJ-PCM-Exo	has_circ_0004658	SMMC-7721/HepG2/Xenograft nude mouse model	Downregulates miR-499b-5p, upregulates JAM3	Inhibits proliferation and induces apoptosis of HCC cells	(54)
M2-EVs	–	Hep3B/HepG2/Tumor xenografts in nude mice	Downregulates MISP, elevates nuclear IQGAP1 levels, activates STAT3 phosphorylation, promotes PD-L1 transcription and expression	Promotes HCC cell immune escape	(241)
M2-THP-1-Exo	miR-501-3p	LC cell lines (SPC-A1, A549, H1299 and H23)/HBE	Targets WDR82	Facilitates proliferation, migration and invasion, and restrains apoptosis of LC cells.	(129)
M1-THP-1-Exo	miR-181a-5p	Human LUAD cell/16HBE/Tumor model in mice	Targets ETS1, inhibits STK16	Inhibits viability and promotes apoptosis in LUAD	(127)
M2-THP-1-Exo	miR-942	LUAD cell lines (SPCA1 and H1299)/HUVECs	Binds to FOXO1 to activate the Wnt/β-catenin pathway	Promotes the migration and invasion of LUAD cells and facilitates angiogenesis	(130)
M2-THP-1-Exo	miR-1911-5p	BEAS-2B/LUAD cell lines (A549 and HCC827)/293T cell line	Targets CELF2, inhibits ZBTB4 expression	Enhances LUAD cell migration and invasion	(131)
M2-THP-1-Exo	miR-3679-5p	A549/293T cell line/Tumor xenografts in nude mice	Reduces NEDD4L-mediated c-Myc ubiquitination	Promotes aerobic glycolysis and induces chemoresistance in lung cancer cells	(135)
M2-THP-1-Exo	lncRNA AGAP2-AS1	Lung epithelial cell BEAS-2B/Human lung cancer cell lines (A549, H157/LTEP-2/NIH-H358/SPCA1)	Downregulates miR-296 and upregulates NOTCH2	Promotes the immunologic function of lung cancer patients after radiotherapy	(136)
M2-BMDM-Exo	miR-155-3p	MB cell lines (Daoy/D283/ONS-76/D341)	Targets WDR82	Promotes MB progression	(133)
M2-BMDM-Exo	miR-588	SGC7901/Xenograft tumor model	Targets CYLD	Contributes to DDP resistance of gastric cancer cells	(20)
M1-PBMC-Exo	miR-16-5p	GC cell lines (AGS and NCI-N87)	Targets PD-L1, activating T cell immune response	Inhibits GC progression	(141)
M2-THP-1-Exo	–	AGS/HGC27 cells	Activates the P38/MAPK signaling pathway	Promotes gastric cancer progression, achieves immune escape	(142)
DCA-RAW264.7-Exo	–	Mouse gastric organoid	Upregulates the expression of TFF2 and GSII	Promotes the development of gastric SPEM	(147)
DCA-THP-1-E	hsa-miR-30a-5p	GES-1	Targets FOXD1, increases CDX2 expression	Promotes intestinal metaplasia and suppresses proliferation of GES-1 cells	(148)
M2-TAMs-Exo	miR-487a	HS-746 T cells	Downregulates TIA1	Promotes the progression of gastric cancer	(143)
M2-BMDM-Exo	miR-21	Gastric cancer cell line (MFC/MGC-803)/Xenograft tumor model	Downregulates PTEN, activates PI3K/AKT pathway	Confers DDP resistance in gastric cancer	(144)
M2-BMDM-Exo	ApoE	MFC/MGC-803	Activates PI3K/AKT signaling pathway	Promotes the migration of gastric cancer cells	(57)
M2-THP-1-Exo	miR-223	GC cell line AGS	Targets FBXW7	Induces the resistance of GC cells to doxorubicin	(145)
M2-PBMC-Exo	lncRNA AFAP1-AS1	KYSE410 cells	Downregulates miR-26a and upregulates ATF2	Promotes the invasion and metastasis of EC	(139)
M2-TAMs-Exo	LINC01592	Eca-109/TE-1/CaES-17 cells/Tumor xenografts in nude mice	Downregulates the expression of MHC-I	Promotes the immune escape of esophageal cancer	(140)
M2-THP-1/MPHs-Exo	miR-365	PDAC K989 cell line/NIH-3T3/Mia PaCa 2	Upregulates the triphospho-nucleotide pool and the enzyme cytidine deaminase in cancer cells	Induces drug resistance in pancreatic adenocarcinoma	(154)
M2-THP-1-Exo	miR-365	PDAC cell lines (PANC‐1, BxPC‐3, MIA Paca‐2 and Capan‐2)/BALB/c male mice	Suppresses BTG2 expression and activates FAK/AKT pathway	Promotes PDAC development	(155)
M2-BMDM-Exo	miR-155-5p/miR-221-5p	MAECs/Tumor-bearing mouse model	Inhibits E2F2 expression	Promotes angiogenesis and growth of pancreatic ductal adenocarcinoma	(153)
M2-THP-1-Exo	miR-501-3p	PDAC cell lines (PANC-1, BxPC-3, MIA Paca-2 and Capan-2)/HMEC-1 PDAC mouse model/	Downregulates TGFBR3, activates the TGF-β signaling pathway	Facilitates the development of PDAC	(161)
M2-THP-1-Exo	miR-21-5p	PC-3/Capan-1/AsPC-1/PANC-1/HPC-Y5/Tumor xenografts in nude mice	Targets KLF3	Stimulates differentiation and activity of PaCa stem cells	(26)
M2-THP-1-EVs	miR-222-3p	PANC-1 cells/Mouse xenograft models of pancreatic cancer cells	Targets TSC1, activatws the mTOR/AKT/PI3K pathway	Promotes chemoresistance of pancreatic cancer	(27)
M2-THP-1-Exo	lncRNA SBF2-AS1	PC cell lines (PANC‐1, BxPC‐3, SW1990, Capan‐2)/Xenograft mouse models	Represses miR‐122‐5p to up‐regulate XIAP	Promotes pancreatic cancer development	(151)
M2-THP-1-Exo	miR-193b-3p	SW1990 cells	Targets TRIM62 that interacts with and induces c-Myc ubiquitination	Promotes the proliferation, migration, invasion, and glutamine uptake of SW1990 cells	(152)
M2-THP-1-EVs	miR-186-5p	SW480/HCT-8	Targets DLC1, activating the β-catenin pathway	Facilitates the growth and motility of CC cells, promotes colon cancer progression	(163)
M2-Exo	miR-155-5p	SW48/CCD841CoN/Nude mouse xenograft model	Downregulates the ZC3H12B expression, promotes IL-6 expression	Promotes immune escape and tumor formation in colon cancer	(160)
M2-THP-1-Exo	miR-183-5p	Human CC cell lines (LoVo/SW480)/Xenograft tumor model	Targets THEM4, activating the PI3K/AKT and NF-κB pathways	Facilitate CC cell proliferation, invasion, and metastasis	(164)
M2-PBMCs-EVs	miR-501-3p	HCT116/FHC/SW480/Tumor xenografts in nude mice	Downregulates the expression of SETD7, upregulates the expression of DNMT1 and SOCS3	Promotes colon cancer progression	(162)
M2-TAM-Exo	miR-21-5p/miR-155-5p	SW48/SW480/CO-115/HUVEC	Downregulates expression of BRG1	Promotes cell migration and invasion in colon cancer	(159)
M2-EVs	miR-143-3p	CRC cell lines (SW480/CCL-228)/NCM460/HUVECs/Xenograft tumor in nude mice	Inhibits ZC3H12A gene and increases C/EBPβ expression	Facilitates the development of CRC	(165)
M2-THP-1-EVs	circRNA_CCDC66	HCT116/FHC/SW480/Tumor xenografts in nude mice	Inhibits miR-342-3p expression, restoring the expression of MTDH	Augments the immune evasion and development of CRC cells	(55)
PMA-THP-1-Exo	miR-503-3p	HCF-10A/BC cell lines/Balb/C nude mice	Targets DACT2 and activates Wnt/β-catenin signaling pathway	Promotes glycolysis and reduces mitochondrial OXPHOS in BC cells	(166)
TAM-EVs	miR-660	Human breast cancer cell lines (MDA-MB-231)	Inhibits KLHL21, activates the NF-κB p65 signaling pathway	Promotes migration and invasion of breast cancer cells	(167)
IL4-TAMs-Exo	miR-223	HEK-293T/SKBR3/MDA-MB-231	Targets the Mef2c-β-catenin pathway	Promotes the invasion of breast cancer cells	(168)
M2-THP-1-Exo	LRRC75A-AS1	Hela cells/Xenograft model	Downregulates miR‐429 expression to elevate SIX1 expression, activates STAT3/MMP‐9 signaling	Promotes cervical cancer progression	(173)
TAM-EVs	GATA3	IOSE80/A2780/SKOV3/OVCAR3/OVCAR8/Tumor xenografts in nude mice	Activates the CD24/Siglec-10 axis	Promotes immune escape and chemotherapy resistance of OC cells	(171)
M2-PBMC-Exo	miR-221-3p	SKOV3 and ID8 cell lines	Targets CDKN1B	Contributes to EOC progression	(170)
TAM-EVs	miR-29a-3p	IOSE80/Human OC cell lines (A2780, SKOV3, ES2, COV504)/OC xenograft models	Targets FOXO3, activates the AKT/GSK3β pathway	Promotes OC cell proliferation and contributes to immune escape	(172)
M2-THP-1-Exo	miR-223	A2780/SKOV3/MCF-7/Xenograft tumor model	Downregulates PTEN, activates PI3K/AKT signaling	Promotes the drug resistance of EOC cells	(169)
M2-THP-hasxo	hsa_circ_0001610	Human EC cell lines Ishikawa/HEC-1B cells/Tumor xenografts in nude mice	Downregulates miR-139-5p and upregulates cyclin B1	Weakens the radiosensitivity of EC cells	(56)
PMA-THP-1/PHM-Exo	–	OSC-4 cells	Activates the AKT/GSK-3β signaling pathway	Decreases the sensitivity to chemotherapeutic agents in OSCC cells	(177)
M2-Exo	miR-31-5p	OSCC cells	Inhibits the Hippo signaling pathway	Facilitates the progression of OSCC	(176)
TAMs-Exo	miR-155-5p	SMCs/IA rat model	Targets GREM1	Promotes IA formation and TAM activation and infiltration	(158)
M2-THP-1-exo	–	IOMM-Lee/CH157-MN	Activates the TGF-β pathway	Promotes the proliferation, migration, and invasion of meningioma cells	(180)
M1-THP-1-Exo	lncRNA HOTTIP	FaDu/Hep-2 cells/Balb/c female nude mice	Activates the TLR5/NF-κB signaling pathway	Suppresses HNSCC progression	(52)
M1-THP-1-Exo		U-251/U-87MG/Brain orthotopic xenografts	Targets MMP-16	Inhibits the proliferation of glioblastoma cells	(175)
M2-THP-1-Exo	miR-15a/miR-92a	Glioma cell lines (T98/U251)	Inhibits PI3K/AKT/mTOR signaling pathway	Inhibits cell migration and invasion of glioma cells	(179)
M2-PBMC-sEV	miR-27a-3p/miR-22-3p/miR-221-3p	GSC 8–11/GSC 20/GSC 267 cells	Targets CHD7, increases molecule RelB as well as phosphorylated STAT3	Induces PMT of GSCs, increases the radioresistance of GSCs	(178)
M2-THP-1-EVs	miR-21-5p	Nthy-ori 3-1/BCPAP/K1/C643/BHT101/HuPBMC-NCG xenograft mice model	Downregulates METTL3 expression, upregulates CD70 protein levels	Promotes immunosuppression, induces resistance to anti-PD-1 therapy	(157)
M1-J774A.1-EVs	miR-29a-3p	B16F10/NIH-3T3/SK-MEL-28	–	Exhibits antiproliferative effects in human melanoma cells	(25)
PCM-Exo	miR-22-3p	human ectopic endometrial stromal cells(eESCs)	Targets SIRT1 and activates NF-κB pathway	Promotes the proliferation, migration, and invasion of eESCs	(22)
PCM-Exo	lncRNA CHL1-AS1	eESCs/EMs mouse model	Downregulates miR-610 and upregulates MDM2	Promotes the proliferation, migration, and invasion of eESCs and inhibits their apoptosis	(21)

#### 2.3.1 Liver-related cancers

Liver cancer is the sixth most prevalent cancer worldwide. Hepatocellular carcinoma (HCC) comprises about 80% of primary liver cancers and is the major pathological type (118). Accumulating evidence suggests that M2-EVs and their components facilitate HCC progression. For example, M2-Exos transmit miR-27a-3p and miR-660-5p to HCC cells, enhancing their proliferation, migration, and invasion by downregulating TXNIP and Krüppel-like factor 3 (KLF3), respectively, while inducing cancer stemness. *In vivo* xenograft models in mice have demonstrated that both M2-Exo miR-27a-3P and miR-660-5p enhance the tumorigenicity of HCC cells (119, 120). M2-EVs promote HCC progression by mediating immunosuppression. In a mouse model of primary hepatocellular carcinoma, M2-EVs promote CD8 + T cell exhaustion via miR-21-5p, accelerating HCC progression. This mechanism involves miR-21-5p promoting CD8+ T cell depletion by targeting YOD1, which activates the YAP/β-catenin pathway (121). M2-EVs also promote HCC progression by promoting tumor angiogenesis. Lu et al. found that M2-Exos augment M2 macrophage recruitment and polarization by promoting CCL2 secretion in HCC cells. M2-Exo miR-23a-3p promotes HCC cell metastasis by targeting PTEN and TJP1 to enhance EMT, angiogenesis, and vascular permeability (122). Additionally, M-Exo miR-92a-2-5p within the tumor microenvironment enhances hepatoma cell invasion by modulating AR and PHLPP/p-AKT/β-catenin signaling (123). However, M-EVs also exhibit an inhibitory effect on HCC development. Aucher et al. showed that macrophages transfer specific endogenous miRNAs (miR-142 and miR-233) into HCC cells in a cell contact-dependent manner, thereby influencing the post-transcriptional regulation of proteins in HCC, decreasing the expression of stathmin-1 (STMN1) and insulin-like growth factor-1 receptor (IGF-1R), and inhibiting HCC cell proliferation (23).

Besides miRNAs, circRNAs secreted by M-Exos are involved in tumor progression. As competitive endogenous RNA, circRNAs competitively bind to miRNAs to affect the expression level of target mRNA, thereby exerting physiological effects. RBP-J modulates TLR-induced inflammatory macrophage polarization through Notch receptor to induce the expression of M1 macrophage-related genes (124). Zhang et al. investigated exosomes from macrophages overexpressing RBP-J and found that hsa_circ_0004658 was upregulated in exosomes, enhancing JAM3 expression by competitively binding to miR-499b-5p, thereby inhibiting HCC cell proliferation and inducing apoptosis (54). These findings suggest that M-EVs in the HCC tumor microenvironment can modulate HCC progression through multiple pathways. HCC is usually diagnosed at an advanced stage; treatment for advanced HCC is limited, and patients have poor prognosis. Thus, exploring M-EVs as potential early biomarkers for HCC is imperative.

#### 2.3.2 Lung-related cancers

Lung cancer (LC) ranks among the most prevalent malignancies globally, characterized by two primary subtypes: small cell lung cancer (SCLC) and non-small cell lung cancer (NSCLC) (125). Lung adenocarcinoma (LUAD) is the predominant variant, representing approximately 40% of all LC cases (126). Numerous studies have elucidated the role of M-Exos in the progression of LC. Specifically, M1-Exo is implicated in the promotion of tumor progression. Serine/threonine kinase 16 (STK16) promotes tumor cell viability and hinders apoptosis through the AKT1 signaling pathway. M1-Exo miR-181a-5p inhibits STK16 by targeting ETS1, thereby diminishing LUAD cells viability, fostering apoptosis, and retarding LUAD progression (127). Several studies have shown that M2-Exos accelerate LC progression by promoting cell migration, invasion, angiogenesis, and suppressing apoptosis (128–131). For example, M2-EV miR-942 promotes migration and invasion of LUAD and angiogenesis by targeting forkhead box (FOX)O1 and activating the Wnt/β-catenin pathway, thereby accelerating the progress of LUAD (130). M2-EV miR-22-3p has been reported to promote osteogenic differentiation of BMSCs by targeting PER2 to activate the Wnt/β-catenin signaling pathway (132). These results indicate that different miRNAs within M2-Exo from the same source act on different target genes and activate the same signaling pathway, thereby exerting physiological effects in different diseases. Another study showed that M2-EV miR-501-3p inhibits apoptosis by targeting WDR82 to promote the proliferation, migration, and invasion of lung cancer cells (129). Although WDR82 is typically underexpressed in tumor tissues, its overexpression in lung cancer cells has been observed to suppress the malignant transformation of these tumor cells. M2-Exo miR-155-3p also enhanced the growth of medulloblastoma cells by targeting WDR82, thus accelerating medulloblastoma progression (133). These results indicate that different miRNAs from the same source of M2-Exos can exert similar physiological effects by acting on the same target gene. The observation that different miRNAs exhibit similar anti-tumor effects on the same target gene suggests that Exos-miRNA primarily serves as an informational molecule. M2-Exos also promote tumor progression by mediating chemoresistance and radiation resistance. Apolipoprotein E (ApoE) secreted by M2-EV worsens the immunogenicity of tumor cells. ApoE inhibits tumor cell immunogenicity by directly binding to and inhibiting BiP function to reduce tumor MHC-I expression, thereby conferring resistance to immunotherapy (134). Wang et al. showed that M2-Exos delivered miR-3679-5p to LC cells and reduced NEDD4L-mediated c-Myc ubiquitination by inhibiting NEDD4L transcription, promoting aerobic glycolysis in LC cells, and inducing chemoresistance (135). These findings provide a basis for studying the mechanisms of drug resistance to improve lung cancer treatment. In addition, Zhang et al. found that lncRNA AGAP2-AS1 is overexpressed in M2-Exos and transferred to LC cells to promote radioresistance in LC cells by downregulating miR-296 and upregulating NOTCH2, the target gene of miR-296. Moreover, the expression levels of lncRNAs AGAP2-AS1 and NOTCH2 were increased in lung cancer cells and tissues, whereas miR-296 levels were diminished (136). This suggests that AGAP2-AS1 serves as a prognostic indicator in patients with LC.

#### 2.3.3 Esophageal and stomach cancers

Esophageal cancer (EC) is among the most lethal malignancies globally, ranking sixth in cancer mortality, primarily composed of squamous cell carcinoma (SCC) and adenocarcinoma (137). Gastric cancer (GC) ranks as the fifth most prevalent cancer and the third leading cause of cancer-related mortality (138). A burgeoning body of research has illuminated the association of M-Exos with tumorigenesis and progression. For example, lncRNA AFAP1-AS1 is highly expressed in M2-Exos and transferred to EC cells by downregulating miR-26a and upregulating ATF2 expression, thus promoting EC invasion and metastasis (139). In addition, M2-Exo LINC01592 accelerates the degradation of MHC-1 and promotes EC immune evasion by activating the E2F6/NBR1/MHC-I signaling pathway. Tumor cells can evade CTL immune attack owing to alterations or defects in MHC-I molecules on the surface of tumor cells (140). M-Exos also affect GC progression by mediating immune escape. M1-Exo deliver miR-16-5p to GC cells and activate the T cell immune response by targeting and inhibiting the expression of PD-L1, thereby reducing immune evasion of GC cells and inhibiting GC progression (141). However, M2-Exos promote the proliferation and migration of GC cells via p38/mitogen-activated protein kinase (MAPK) signaling pathways and achieve immune evasion by increasing the expression of PD-L1, thereby promoting GC progression (142). Additionally, M2-Exo miR-487a promotes GC proliferation by inhibiting the expression of the tumor suppressor gene T-lymphocyte antigen 1 (TIA1) (143). Multiple studies have shown that M2-Exos plays an important role in GC progression and chemoresistance (20, 144, 145). Chemotherapeutic agents such as cisplatin (DDP), doxorubicin (DOX), and paclitaxel (PTX) are used to treat various malignancies, including GC (146). Cui et al. showed that M2-Exo miR-588 contributes to DDP resistance in gastric cancer cells by partially targeting CYLD (20). Similarly, M2-Exo miR-21 is transferred to GC cells and downregulates PTEN to activate the PI3K/AKT signaling pathway, reducing chemosensitivity and apoptosis in GC cells and conferring DDP resistance in gastric cancer (144). In another study, M2-Exo delivered miR-223 to GC cells and induced DOX resistance in GC cells by targeting F-box and WD repeat domain-containing 7 (FBXW7). Moreover, the expression of plasma exosomal miR-223 is significantly increased in patients with GC with DOX-resistance, suggesting that miR-223 may be a potential indicator of DOX resistance in gastric cancer (145). Studies have shown that DCA-induced M-Exos indirectly affect GC progression. Xu et al. found that deoxycholic acid-stimulated M-Exos promoted the development of antispasmodic polypeptide-expressing metaplasia (SPEM) in mouse gastric organoids. SPEM is an important risk factor for gastric cancer (147). Similarly, deoxycholic acid-induced M-Exos were enriched with high levels of hsa-miR-30a-5p, and the overexpression of hsa-miR-30a-5p inhibited gastric epithelial cell proliferation and promoted intestinal metaplasia by targeting FOXD1. Gastrointestinal metaplasia is considered to be a risk factor for enteral gastric cancer (148). M2-Exos also exert physiological functions in GC by delivering proteins. For example, M2-Exos transfer functional ApoE into GC cells and promote GC cell migration by activating the PI3K-Akt signaling pathway (57).

#### 2.3.4 Pancreas-related cancers

Pancreatic cancer (PC) is a highly lethal malignancy that ranks seventh among cancer-related mortalities worldwide (149). Pancreatic ductal adenocarcinoma (PDAC) is the most common type of PC, accounting for >85% of all PC cases (150). M-Exo miRNAs and lncRNAs also affect PC progression. The lncRNA SBF1-AS2 is significantly upregulated in M2-Exos and upregulates the expression of XIAP by inhibiting the expression of miR-122-5p, thereby promoting the tumorigenicity of PC cells (151). Both miR-21a-5p and miR-501-3p are derived from M2-Exos and have been confirmed to promote PC cell migration and invasion (26, 152). The microRNA miR-21a-5p is up-regulated in M2-Exos and transferred to human pancreatic cancer cells (PaCa) to regulate the differentiation and activity of PaCa stem cells by directly targeting KLF3 (26). However, miR-501-3p promotes the migration, invasion, and tube formation of PDAC cells by targeting the TGFβR3 gene to activate the TGFβ signaling pathway, thus promoting the progression of PDAC (152). In addition, M2-Exo miR-155-5p and miR-221-5p have been confirmed to promote angiogenesis and subcutaneous tumor growth in PDAC by targeting E2F2 (153). M2-exo also influences PC development by mediating chemoresistance. Gemcitabine is the first-line chemotherapeutic drug for the treatment of PC; however, drug resistance limits the efficacy of chemotherapy. Guo et al. found that miR-222-3p increases cell proliferation and angiogenesis and decreases apoptosis in the tumor tissues of mice exposed to gemcitabine, mainly through the mechanism of miR-222-3p-induced gemcitabine resistance in PC progression by targeting TSC1 and activating the mTOR/AKT/PI3K pathway (27). Similarly, Binenbaum et al. found that M2-Exo miR-365 impairs gemcitabine activation by upregulating the nucleotide pool of triphosphates in cancer cells and inducing cytidine deaminase, thereby significantly reducing the sensitivity of PDAC cells to gemcitabine (154). Another study showed that M2-Exo miR-365 promoted the migration, invasion and tube formation of PDAC cells by targeting the TGFβR3 gene to activate the TGFβ signaling pathway, thus promoting the progression of PDAC (155). This suggests that the same miRNAs from the same source of M-Exos can exert similar physiological effects on diseases through different pathways. M2-Exo miR-3679-5p reduces NEDD4L-mediated c-Myc ubiquitination and promotes LC progression (135). Zhang et al. found that M2-Exo miR-193b-3p reduces TRIM62-mediated c-Myc ubiquitination by targeting TRIM62, and promotes the proliferation, migration, invasion, and glutamate uptake of PC cells *in vivo* and *in vitro*, thus promoting PC progression (152).

#### 2.3.5 Colon-related cancers

Colon cancer (CC) ranks among the most prevalent malignancies within the digestive tract and constitutes the third leading cause of cancer-related mortality globally (156). Many M2-EV miRNAs have been found to be involved in CC development. MiR-21-5p is a tumorigenic miRNA targeting KLF3, YOD1, and METTL3, thus promoting pancreatic, hepatocarcinoma, and thyroid cancer progression and inducing chemoresistance (26, 121, 157). Likewise, miR-155-5p is confirmed to be tumorigenic, targeting GREM1 and E2F2, thereby promoting intracranial aneurysms formation and angiogenesis in pancreatic ductal adenocarcinoma (153, 158). Lan et al. found that miR-21-5p and miR-155-5p are upregulated in M2-Exos and transferred to colorectal cancer cells, facilitating their migration and invasion by targeting and downregulating BRG1 expression (159). In addition, M2-Exo miR-155-5p enhances IL-6 expression by inhibiting ZC3H12B, thereby promoting immune escape and tumorigenesis in CC (160). This further illustrates that M2-Exo miR-21-5p and miR-155-5p have protumor activity and can promote the progression of multiple cancers. M2-Exo miR-501-3p promotes lung cancer and PDAC progression by targeting WDR82 and TGFβR3 (129, 161). Ding et al. found that miR-501-3p was transferred to CC cells via M2-EVs, targeting and downregulating SETD7, thereby upregulating DNMT1 and SOCS3, which enhances the malignant signature of CC cells *in vitro* and tumor growth *in vivo* (162). This suggests that identical miRNAs from common sources may exert analogous roles across diverse tumors. Studies have shown that M2-EV miRNAs can also promote CC progression by mediating inflammation-related signaling pathways. Both miR-186-5p and miR-183-5p are upregulated in M2-EVs and can promote the proliferation, invasion, and metastasis of CC cells by targeting DLC1 and THEM4, respectively, activating the β-catenin, PI3K/AKT, and NF-κB signaling pathways, thereby accelerating the progression of CC (163, 164). Another study reported that M2-EVs delivered miR-143-3p to colorectal cancer cells, where it promotes CC development by targeting ZC3H12A to upregulate C/EBP-β expression (165). Beyond miRNAs and lncRNAs, circRNAs secreted by M-EVs contribute to CC development. CircRNAs are a highly abundant and heterogeneous class of non-coding RNAs enriched in EVs. Fan et al. reported that M2-EVs enhance CC cell immune escape by delivering circRNACCDC66, inhibiting miR-342-3p expression and upregulating MTDH expression, thereby promoting CC development (55). Nevertheless, the physiological impacts of M-EV circRNAs in diseases remain underexplored.

#### 2.3.6 Female-related cancers

Recent studies have shown that TAM-EVs are involved in the progression of some female-related cancers such as breast, cervical, ovarian, and endometrial cancers. Breast cancer (BC) is one of the most common malignancies in women. Studies have shown that miR-503-3p is overexpressed in TAM-EVs, activating the Wnt/β-catenin signaling pathway by targeting DACT2, promoting glycolysis and reducing mitochondrial oxidative phosphorylation in BC cells, thereby advancing BC development (166). Aberrant activation of the Wnt/β-catenin signaling pathway mediated by TAM-EVs has associated with several cancer types, including PDAC and CC (130, 163). BC metastasis is the leading cause of cancer-related death in women worldwide. Studies have shown that TAM-EV miRNAs promote the invasion and migration of BC cells. For example, miR-660 overexpressed in TAM-EVs, is transferred to BC cells, where it promotes invasion and migration by inhibiting KLHL21 and activating the NF-κB signaling pathway (167). In a similar study, M2-Exos shuttle miR-223 into BC cells and promotes BC cell invasion through the Mef2c-β-catenin pathway (168). These findings offer novel insights for developing treatments targeting breast cancer metastasis. Another study showed that miR-223 induces drug resistance in epithelial ovarian cancer (EOC) cells by targeting PTEN to activate the PI3K/AKT signaling pathway and trigger a chemoresistant phenotype (169). TAM-EV miR-21 also induces chemotherapy resistance in gastric cancer cells by targeting PTEN to activate the PI3K/AKT signaling pathway (144). These results indicate that different miRNAs can mediate the same signaling pathway by targeting the same target gene to exert similar physiological effects, further revealing that multiple miRNAs play a synergistic role in the development of diseases, rather than relying on a single miRNA. In addition to EOC and GC, TAM-EV-mediated aberrant activation of the PI3K/AKT pathway drives the progression of various cancers, including PC and CC (27, 164). In EOC, TAM-EV miR-221-3p exerts oncogenic effects by targeting CDKN1B, thereby regulating proliferation, migration, and G1/S transformation of EOC cells (170).

Ovarian cancer (OC) is a malignancy impacting the female reproductive tract. TAM-EVs facilitate OC progression by mediating immune escape and chemotherapy resistance. Chen et al. reported GATA-binding protein-3 (GATA3) expression in TAM-EVs. GATA3 is transferred into OC cells via TAM-EVs, inhibiting T-cell activity through the CD24/Siglec-10 pathway, thus promoting immune escape and chemoresistance (171). In addition, TAM-EV miR-29a-3p enhances PD-L1 expression by mediating the FOXO3-AKT/GSK3β axis, thereby promoting OC cell proliferation and immune escape *in vitro* and *in vitro* (172). These findings may provide novel therapeutic targets for the treatment of OC.

Cervical cancer is the leading malignant cancer in women worldwide, and studies have shown that TAM-EV lncRNAs are involved in the progression of cervical cancer. LRRC75A-AS1 promotes proliferation, migration, invasion, and EMT in cervical cancer by inhibiting miR-429 expression, upregulating SIX1 expression, and activating the signal transducer and activator of transcription (STAT)3/MMP-9 signaling axis (173). Endometrial cancer is a common gynecological malignancy that seriously threatens women’s health. Cyclin B1 is an important promoter that induces radioresistance in cancer cells by regulating the cell cycle. Studies have shown that hsa _circ_0001610 is overexpressed in M2-Exos and transferred to endometrial cancer cells, and negatively regulates the expression of miR-139-5p in endometrial cancer cells by directly binding to miR-139-5p, upregulating the expression of cyclin B1, thereby attenuating the radiosensitivity of endometrial cancer cells (56). Endometriosis (EMS) is a debilitating disease with a chronic inflammatory profile that resembles malignancy and includes progressive and aggressive growth (174). Studies have shown that peritoneal macrophage-derived exosomes (PCM-Exos) influence EMS progression PCM-Exo miR-22-3p promotes the proliferation, migration, and invasion of human ectopic endometrial stromal cells (eESCs) by targeting SIRT1 and activating the NF-κB pathway (22). Another study showed that PCM-Exos promoted the proliferation, migration, and invasion of eESCs by transporting lncRNA CHL1-AS1 into eESCs to downregulate miR-610 and upregulate MDM2 (21).

#### 2.3.7 Other cancers

TAM-EVs also affect the progression of other types of cancer such as oral squamous cell carcinoma, head and neck squamous cell carcinoma, intracranial aneurysm, meningioma, glioma, and medulloblastoma. M1-Exo have inhibitory effects on tumors, including head squamous cell carcinoma (HNSCC), glioma, and human melanoma. The lncRNA HOTTIP is a key molecule in M1-Exo and activates the TLR5/NF-κB signaling pathway by competitively binding to miR-5a-19p and miR-3b-19p, thereby inhibiting the progression of HNSCC (52). M1-Exo miR-150 inhibits glioma progression by targeting MMP-16 to inhibit human glioma cells (175). However, M1-EV miR-29a-3p inhibits the proliferation of human melanoma cells and exerts antitumor effects (25). In oral squamous cell carcinoma (OSCC), M2-Exo miR-31-5p downregulates the expression of the tumor suppressor gene LATS2 by inhibiting the Hippo signaling pathway, thereby promoting OSCC progression (176). In addition, M-Exos attenuate the effects of chemotherapeutic agents on cell cycle regulation and apoptosis by activating the AKT/GSK-3β signaling pathway, thereby reducing the sensitivity of OSCC cells to the chemotherapeutic agents 5-fluorouracil (5-FU) and cis-diamine diachloroplatin (CDDP) (177). M2-EVs also mediate immunosuppression and chemoresistance in thyroid cancer. Ning et al. showed that M2-EV miR-21-5p downregulated METTL3 expression in thyroid cancer cells, thereby leading to demethylation and stabilization of CD70 mRNA in a YTHDF2-dependent manner and that upregulation of CD70 protein levels increases the abundance of immunosuppressive Tregs and terminal failure T cells, thus promoting immunosuppressive and anti-PD-1 therapy resistance (157). In addition, M2-EVs induce drug resistance in glioma cells. The expression of miR-27a-3p, miR-22-3p, and miR-221-3p is significantly upregulated in M2-EVs; these miRNAs promote neuromesenchymal transition (PMT) and radioresistance in glioma stem cells by targeting CHD7 to increase RelB-P50 and phosphorylated STAT3 (178). Yao et al. found that miR-15a and miR-92a blocks the PI3K/AKT/mTOR signaling pathway and inhibits glioma cell invasion and migration by binding to CCND1 and RAP1B, respectively. M2-Exos with insufficient miR-15a and miR-92a expression promote the migration and invasion of glioma cells (179). Another study showed that M2-Exos contribute to the development of meningioma by activating the TGFβ signaling pathway to promote the proliferation, migration, and invasion of meningioma cells (180). TAM-Exos can be absorbed by tumor cells as well as other cells, thereby regulating tumor progression. Studies have shown that miR-155-5p is upregulated in TAM-Exos and transported to smooth muscle cells (SMCs) to promote the proliferation and migration of SMCs by targeting GREM1, thereby promoting the formation of intracranial aneurysms and the activation and infiltration of TAMs (158).

### 2.4 Inflammatory diseases

EVs derived from macrophages of different phenotypes and their contents have different effects on the occurrence and development of inflammatory diseases, depending on their parental cell types and the cargo they carry (**Table 4**). In experimental autoimmune neuritis (EAN) animal models, M1 macrophage-derived exosomes (M1-Exo) directly regulate T cells, enhance Th1 cell differentiation (increase proportion) and effector function (increase IFN-γ intensity), and increase IFN-γ production in CD8+ T cells, promoting EAN progression (181). M2 macrophage-derived exosomes (M2-Exo) showed the potential to attenuate EAN. M-EVs lacking the endonuclease XPF-ERCC1 induce persistent DNA damage, promote cellular glucose uptake, and activate innate immune responses, leading to increased inflammation (182). In this procedure, Irreparable DNA damage triggers an exosome-based, metabolic reprogramming that leads to chronic inflammation and tissue pathology in nucleotide excision repair progeroid syndromes. Anti-inflammatory miRNAs (miR-146a, miR-146b, and miR-21-3p) in small EVs (sEVs) from LPS-stimulated RAW264.7 macrophages (LPS-M-sEV) downregulate pro-inflammatory target genes. In a mouse model of Freund’s-adjuvant-induced inflammatory pain, LPS-M-sEVs alleviated pain by modulating multiple genes (24). In the same animal model, M2-EVs miR-23a-3p modulated the histone deacetylase 2/nuclear factor erythroid 2–related factor 2 axis by targeting ubiquitin-specific proteinase 5 expression to reduce inflammatory pain (183). These results highlight the role of M-EVs in mediating inflammation and immune responses.

**Table 4:** Summary of the biological functions of M-EVs in inflammatory diseases.

EV source	Effective molecules	Model system	Mechanism	Biological function	Reference
M1-BMDM-Exo	–	Splenic T cells/Splenic MNCs/EAN rat model	Boosts Th1 and Th17 response	Aggravates experimental autoimmune neuritis	(181)
LPS-BMDM-Exo	miR-223	Colon organoid/IBD mouse model	Inhibits TMIGD1	Promotes intestinal mucosal barrier dysfunction in inflammatory bowel disease	(185)
M2-THP-1-Exo	miR-590-3p	FHC cells/DSS-induced colitis mouse model	Targets LATS1, activates YAP/β-catenin-regulated transcription	Reduces inflammatory signals and promotes epithelial regeneration	(186)
RAW264.7-sEV	Lactadherin	IEC-6 cells/Ptpn1-knockout mice	Induces M2 macrophage polarization, suppresses TNF-α and NF-κB signaling	Alleviates intestinal inflammation	(187)
LPS-RAW264.7-EVs	miR-146a/miR-146b/miR-21	Primary neuronal/Glial cells/Inflammatory pain mouse model	Decreases pro-inflammatory gene expression	Attenuates mechanical allodynia	(24)
AAI-THP-1-EVs	LRG1	HK-2/mTECs/AAN mouse model	Depends on TGFβR1 signaling	Promotes oxidative stress, induces TEC injury	(242)
M2-BMDM-EVs	–	CIA animal model	Guides *in situ* macrophage reprogramming toward anti-inflammatory M2 macrophages	Ameliorate synovial inflammation and protects against joint destruction in CIA mice	(243)
M2-BMDM-Exo	miR-370	ASMCs/Asthma mouse model	Suppresses FGF1 expression and the MAPK/STAT1 signaling pathway	Alleviates asthma progression	(244)
M2-EVs	M2-EV-Gel Ink	HUVECs/Mouse skin defect model	Reprograms macrophage polarization and promotes the proliferation and migration of endothelial cells	Regulates inflammation and enhances angiogenesis in wounds	(245)
M1-RAW264.7-EVs	–	ESC/HUVECs/Mouse endometriosis model	Inhibits the migration and invasion of EM-ESCs and reprograms M2 macrophages	Suppresses the development of endometriosis	(196)
M1-PCM-Exo	miR-21a-5p	FHC cells	Targets E‐cadherin, promotes ILC2 activation	Aggravates DSS‐induced enteritis	(184)
M2-BMDM-ABs	miR-21-5p	NIH-3T3 fibroblasts/HUVECs/Mouse knee OA model	Activates M1-Mφs into a M2-like phenotype	Alleviates the progression of OA	(29)
M2-RAW264.7-EVs	miR-23a-3p	Primary microglia/Inflammatory pain mouse model	Targets USP5, regulates the HDAC2/NRF2 axis	Alleviates inflammatory pain	(183)
PMA-THP-1-EVs	–	Placental villous explants	Increases IL-6, IL-8, IL-1ß and TNF-α expression	Induces placental pro-inflammatory responses	(195)
RAW264.7-Exo	miR-155	Primary rat cardiomyocyte/Uremic mouse model	Targets FoxO3a	Promotes cardiomyocyte pyroptosis and uremic cardiomyopathy changes in uremic mice	(246)
M2-PCM-EVs	lncRNA MEG3	Murine colon epithelial cell/YAMC/UC murine model	Up-regulates MEG3 expression and promotes CREB1 transcription	Enhances cell viability and reduces inflammatory responses in UC	(53)
PMA-THP-1-Exo	–	AAA mouse model	Activates JNK and p38 pathways, Increases MMP-2 expression	Contributes to the development of AAA	(192)
M2-BMDM-EVs	miRNA	Super- or naturally ovulated mouse models/Aged mice	Downregulates the mTOR signaling pathway	Ameliorates the inflammatory microenvironment within the ovary and improves ovarian function in aged mice.	(197)
PCM-Exo	–	BV2 cells/Rat spinal cord contusion model	Downregulates the PI3K/AKT/mTOR signaling pathway	Activates and promotes autophagy in microglia, increases anti-inflammatory type microglial polarization	(191)

MiR-21a-5p is elevated in peritoneal M-Exo of dextran sulfate (DSS)-induced ulcerative colitis (UC) mice and promotes innate lymphoid type-2 cell activation by decreasing E-cadherin expression, leading to intestinal mucosal epithelium damage and worsened enteritis (184). In a DSS-induced inflammatory bowel disease model, LPS-stimulated bone marrow-derived M-Exo miR-223 accelerated colitis by inhibiting transmembrane and immunoglobulin domain-containing protein 1 to induce intestinal barrier dysfunction (185). Conversely, M2-Exo miR-590-3p suppressed pro-inflammatory cytokines by targeting large tumor suppressor kinase 1, activating yes-associated protein (YAP)/β-catenin-regulated transcription, thereby reducing inflammatory signaling, accelerating epithelial repair and wound healing in DSS-induced colitis (186). M2-EVs protect against intestinal inflammation; they upregulate maternally expressed gene 3 (MEG3) by delivering lncRNA MEG3 to colonic epithelial cells, then promoting the transcription of CAMP responsive element binding protein 1 via competitive binding to miR-20b-5p, thereby enhancing cell viability and mitigating inflammatory response in UC (53). The sEVs from tyrosine-protein phosphatase non-receptor type 1-knockout macrophages also alleviate intestinal inflammation, mainly by inducing macrophage M2 polarization and inhibiting TNF-α and nuclear factor kappa B (NF-κB) signaling via transporting mucin-containing cargo (80, 187).

In a mouse model of cancer-induced bone pain (CIBP), M2-Exo exerts antinociceptive effects by delivering miR-216a, which targets high mobility group box 1 expression to downregulate the TLR4-NF-κB signaling pathway (188). M2 macrophage-derived apoptotic bodies can prevent or alleviate osteoarthritis (OA) by reprogramming M1 to M2 macrophages, thus reducing chondrocyte damage (29). In a rat model of knee osteoarthritis (KOA), M2-EVs reduced cartilage inflammation by modulating the PI3K/AKT/mTOR pathway (189). Studies indicate that PI3K/AKT/mTOR pathway activation exerts anti-arthritic effects by enhancing chondrocyte proliferation and reducing apoptosis (190). In a rat model of spinal cord injury, peritoneal macrophage-derived exosomes (PCM-Exos) activated and promoted microglial autophagy by downregulating the PI3K/AKT/mTOR signaling pathway, thereby increasing anti-inflammatory microglial polarization, stimulating local microglial anti-inflammatory properties, and promoting post-spinal cord injury repair (191).

Abdominal aortic aneurysm (AAA) is a chronic inflammatory disease with no effective treatment currently available. Wang et al. demonstrated that M-Exos participate in AAA pathogenesis by activating the c-Jun N-terminal kinase (JNK) and p38 pathways to induce matrix metalloproteinase (MMP)-2 production (192). This suggests that M-Exos serve as a therapeutic target, warranting further investigation. Hemorrhagic shock (HS) predisposes patients to systemic inflammatory syndromes. *In vivo* and *in vitro* HS models have shown that the exosomes from HS-activated alveolar macrophages promote polymorphonuclear neutrophil (PMN) necroptosis by upregulating the production of nicotinamide adenine dinucleotide phosphate (NADPH) oxidase-derived ROS in PMNs, subsequently enhancing inflammation and tissue damage (193). Previous studies showed that M-EVs affect women’s health by modulating inflammatory factor expression. M-EVs enter the placenta via clathrin endocytosis, inducing pro-inflammatory responses and modulating placental cytokine production, thus potentially promotes responses to maternal inflammation and infection, thereby preventing fetal injury (194, 195). This may reveal a new way of transmitting information between the mother and placenta. M1-EVs are associated with pro-inflammatory and harmful effects but also have protective roles. M1-EVs suppress the migration and invasion of endometriosis-eutopic endometrial stroma cells (EM-ESCs) and hinder endometriosis development by reprogramming M2 to M1 macrophages, thereby reducing human umbilical vein endothelial cells (HUVEC) tube formation (196). These findings suggest a potential strategy for the treatment of endometriosis. Another study showed that M2-EVs contain specific miRNAs that improve ovarian function in elderly mice by downregulating the mTOR signaling pathway and improving the inflammatory microenvironment within the ovaries (197). These results suggest that M-EVs are crucial in inflammatory diseases, paving the way for clinical applications.

### 2.5 Bone-related diseases

Macrophages are crucial for bone homeostasis (198, 199). Numerous studies indicate that M-EVs are pivotal in bone diseases like fractures, osteoarthritis, osteoporosis, and tendon injury. M-EVs mainly mediate osteoblast differentiation, angiogenesis, immune cell reprogramming, cell proliferation, and apoptosis (**Table 5**). Mesenchymal stem cell (MSC) osteogenic differentiation is a key factor in fracture healing (198). M1-Exos upregulate IL-6 and TNF-α in BMSCs, thus inhibiting their osteogenic differentiation through inflammatory mediation (200). Xia et al. investigated how different macrophage-derived exosomes (M0/M1/M2-Exos) affect BMMSC proliferation, osteogenesis, and adipose differentiation, finding that M1-Exos enhance BMMSC proliferation. M1-Exos have a more pronounced effect on BMMSC proliferation and osteogenic differentiation compared to M0-Exos or M2-Exos (201). Moreover, M1-Exos promote the osteogenic differentiation of BMSCs through microRNA-21a-5p during early inflammation (202). This seems to contradict previous results, but it is an interesting possibility that M-Exos of different phenotypes promote the osteogenic differentiation of BMSCs at different stages of the disease. This is consistent with the fact that M0 macrophages support osteogenesis during normal bone homeostasis, whereas M1 and M2 macrophages contribute to different stages of fracture healing (198). Angiogenesis is a prerequisite for osteogenesis during bone repair and regeneration. *In vitro* studies have shown that Mg^2+^-stimulated M-Exos inhibit angiogenesis in endothelial cells by down-regulating endothelial nitric oxide and vascular endothelial growth factor (203). However, exosomes derived from macrophages upon copper or cobalt ions promote angiogenesis (204, 205). M1-Exos also impact bone loss and osteoporosis. In a mouse model of postmenopausal osteoporosis, M1-Exo miR-98 exacerbated bone loss and osteoporosis by targeting DUSP1 and activating the JNK signaling pathway (205). In contrast, miR-690-enriched M2-Exos promote BMSC osteogenesis and reduce lipogenesis by upregulating IRS-1 and TZ levels, potentially offering a treatment for bone loss (207). M2-Exos also promote osteogenic differentiation of BMSCs via multiple pathways, exerting osteoprotective effects (48, 132, 208, 209). For example, M2-Exo miRNA-26a-5p promotes expression of osteogenic differentiation-related proteins ALP, RUNX-2, OPN, and Col-2, induces BMSCs osteogenic differentiation, and inhibits adipogenic differentiation (208). M2-Exo miR-22-3p promotes BMSCs osteogenic differentiation by targeting PER2 to activate the Wnt/β-catenin signaling pathway (132). In a mouse fracture model, M2-Exos inhibited the expression of salt-induced kinases 2 and 3 (SIK2 and SIK3) by delivering miR-5106 to promote osteogenic differentiation of BMSCs and accelerate fracture healing (209). The anti-inflammatory cytokine IL-10 is a key regulator in bone remodeling (198). M2-Exos deliver IL-10 mRNA directly to BMSCs and BMDMs, activating the cellular IL-10/IL-10R pathway, thereby promoting BMSC osteogenic differentiation and inhibiting BMDM formation (48). Mg^2+^ promotes osteogenesis and prevents inflammation, with Mg^2+^-induced M-Exos enhancing BMSC osteogenic differentiation by downregulating miR-381 (210). In bone tissue regeneration and repair, macrophage-derived small extracellular vesicles (ICM-M-sEV) treated with biomimetic intrafibrous mineralized collagen (IMC) promote osteogenic differentiation of BMSCs through the BMP2/Smad5 pathway (211). M2-Exos play a role in periodontal tissue regeneration and bone remodeling and enhance osteogenic differentiation of human periodontal ligament stem cells (PDLSC) (212). M2-Exos also induce healing of muscle and tendon injuries. The miR-501-enriched M2-Exo targeting YY1 promotes myoblast differentiation, myotube formation, and regeneration of damaged pubococcygeal muscle (213). M2-Exo miR-21-5p enhances tendon cell proliferation, migration, and fibrosis by suppressing Smad7, thus inducing peritendon fibrotic healing after tendon injury *in vivo* (214). Osteoarthritis (OA) is an inflammation-mediated patholytic disease closely linked to cartilage deformity severity. M1-EVs advance OA pathogenesis by activating atypical pyroptosis in chondrocytes and exacerbating chondrocyte catabolism and cartilage degradation (215).

**Table 5:** Summary of the biological functions of M-EVs in bone-related diseases.

EV source	Effective molecules	Model system	Mechanism	Biological function	Reference
M2-BMDM-Exo	miR-26a-5p	BMSCs	Upregulates the expression of ALP, RUNX-2, OPN, and Col-2	Promotes the osteogenesis differentiation of BMSCs	(208)
M2-BMDM-Exo	IL-10 mRNA	BMSCs/BMDM/Murine periodontal disease model	Activates the cellular IL-10/IL-10R pathway	Promotes BMSC osteogenic differentiation, suppresses BMDM osteoclast formation	(48)
BMDM-Exo	miR-21-5p	NIH 3T3/Tenocytes	Inhibits the expression of Smad7	Promotes fibrogensis of tendon cells, induces fibrous tissue formation	(214)
M2-BMDM-Exo	–	KOA rat -model	Downregulates the expression of PI3K/AKT/mTOR	Attenuates the inflammatory response and pathological damage of articular cartilage in KOA rats	(189)
LPS-PMCs-EVs	–	Primary murine chondrocytes/ACLT-induced OA models	Activates the caspase 4/caspase 11–GSDMD pathway, induces chondrocyte pyroptosis	Promotes cartilage catabolism and the development of OA	(215)
M2-RAW264.7-Exo	miR-690	Primary BMSCs	Increases the expression of IRS-1 and TAZ	Promotes osteogenic differentiation of BMSCs	(207)
M2-THP-1-Exo	–	Primary hPDLSCs	Upregulates the expression of ALP and OCN proteins	Promotes osteogenic differentiation of human PDLSCs	(212)
IMC-THP-1-sEV	–	MSCs	Activates the BMP2/Smad5 pathway	Facilitates MSC osteogenesis	(211)
M1-RAW264.7-Exo	miR-21a-5p	BMSC	Upregulates the expression of osteogenic genes ALP, OPN, BMP2, Runx2 and COL-1	Promotes osteogenesis of BMSCs	(202)
LPS-RAW264.7-Exo	–	BMSC	Mediates inflammatory stimulation	Inhibits BMSC osteogenic differentiation	(200)
M1/M2-SPMs-Exo	lncRNA (LOC103691165)	BMSCs/Rat tibia fracture model	Upregulates the expression of osteogenic genes OPN, BMP2, Runx2, and CO	Promotes BMSC osteogenesis	(247)
M1-RAW264.7-Exo	–	BMMSCs	Upregulates the expression of BMP2, OCN, and Runx2	Promotes osteogenesis of BMMSCs	(201)
M2-BMDM-Exo	miR-5106	BMSCs/Mouse fracture model	Suppresses the expression of SIK2 and SIK3	Promotes osteogenic differentiation of BMSC, accelerates fracture healing	(209)
M1-RAW263.7-Exo	miR-98	MC3T3-E1 cells/Murine model of OP	Targets DUSP1, activates the JNK signaling pathway	Exacerbates bone loss and osteoporosis	(206)
M2-RAW264.7-Exo	miR-501	C2C12/SUI mouse model	Targets YY1	Promotes myoblast differentiation, improves the injured pubococcygeal muscle	(213)
Mg2+-PMCs-Exo	miR-381	BMSCs	Downregulates the expression of ALP, Runx2	Suppresses the osteogenic differentiation of BMSCs	(210)
M2-THP-1-EVs	miR-22-3p	Human BMSCs/AS mouse model	Targets PER2, activating the Wnt/β-catenin signaling pathway	Induces the osteogenic differentiation of BMSCs in AS	(132)
CuSO4- MΦs-Exo	–	EC	Mediates the trafficking of integrin β1 of Ecs to recycling endosomes	Promotes angiogenesis of endothelial cells	(204)
Co-RAW264.7-Exo	–	EC	Upregulates NO, VEGF, and integrin β1 expression of Ecs	Promotes angiogenesis	(205)

### 2.6 M-EVs as drug-delivery systems

Many studies have shown that M-EVs serve as drug delivery systems enhancing efficacy owing to their excellent stability, biosafety, compatibility and ability to cross the blood-brain barrier (BBB). M-EVs can be engineered by incorporating low-molecular-weight chemotherapeutic drugs, such as PTX and DOX, or therapeutic proteins, such as catalase and brain-derived neurotrophic factor, into EVs through method like co-incubation, electroporation, saponin permeabilization, freeze-thaw cycles, or sonication (216–218). Engineered M-EVs, as new drug delivery platforms, have several advantages over traditional delivery methods, including high biocompatibility, enhanced stability, precise targeting, and low immunogenicity (219). M1-EVs alone showed antitumor effects, as previously described (52, 127, 141). When M1-EVs serve as drug carriers alongside other drugs, they significantly improve the antitumor efficacy. For example, M1-EVs loaded with PTX, DOX, DDP, docetaxel (DTX), gemcitabine (GEM), and deferasirox (DFX) significantly improve the antitumor effects of these chemotherapeutic agents *in vitro* and *in vivo* by inhibiting tumor cell growth or inducing mitochondrial dysfunction, preventing M1 macrophages from repolarizing to the M2 phenotype, or increasing their sensitivity (216, 220–225). Genetic and chemical modifications can augment engineered M-EVs’ targeting and efficacy in tumor therapy. Kim et al. developed and optimized a formulation of PTX-loaded exosomes with an aminoethylanisamide-polyethylene glycol (AA-PEG) vector moiety to target the sigma receptor overexpressed in lung cancer cells. PTX-loaded AA-PEG-vectored exosomes (AA-PEG-exoPTX) have high loading capacity and targeting ability, enhancing antitumor efficacy upon systemic administration (226). Li et al. modified M-EVs with peptides targeting mesenchymal-epithelial transition factors, creating an M-EV-coated polylactic acid-glycolic acid copolymer nanoplatform that enhanced DOX’s cellular uptake and antitumor efficacy (227). Zhang et al. created a tri-modal synergistic therapy with exosome-based nanoplatforms. Utilizing exosomes as carriers, the UN@mSiO2-Ce6 (UC) probe was integrated into M1-Exos, forming the M1UC probe. The M1UC probe not only reprograms immunosuppressive M2 macrophages into antitumor M1 macrophages for immunotherapy but also catalyzes the endogenous production of nitric oxide (NO) by iNOS for gas therapy. Simultaneously, NO can react with ROS produced by the photosensitizer Ce6 to generate peroxynitrite, significantly enhancing the lethality of PDT in tumors (228). Engineered M-EVs mitigate inflammatory disease progression by targeting inflammation areas and enhancing anti-inflammatory effects. Studies have shown that loading the anti-inflammatory drug mycophenolic acid (MPA) into M-EVs as an immunomodulatory nanoplatform significantly enhanced the anti-inflammatory and antioxidant effects of MPA *in vitro* compared with those of free drugs (229). Simultaneously, loading melatonin into M-EVs specifically targets inflammatory areas and mediates immune reprogramming, thereby polarizing macrophages from M1 to M2 phenotypes (230). In addition, Wu et al. loaded 5-aminolevulinate hexyl hydrochloride (HAL) into M2-Exos via electroporation, leading to intrinsic heme biosynthesis and metabolism to produce anti-inflammatory carbon monoxide and bilirubin, enhancing anti-inflammatory effects (231). Moreover, Cheng et al. loaded stereoscopic Pd into M-EVs to construct a biomimetic nanoformulation (Pd-M-EVs). Pd-M-EVs inhibit macrophage M1 polarization and reduce neutrophil infiltration and recruitment by blocking mTORC1-HIF-1α-mediated glycolysis, thereby promoting colonic inflammation and remodeling of the immune microenvironment, improving the barrier function of the colonic mucosa, and effectively alleviating UC (232). Engineered M-EVs have also been used in fracture healing. Shou et al. conjugated 3WJ RNA nanoparticles with BMSC adapters to M2-Exos, creating aptamer-functionalization exosomes (3WJ-BMSCapt/M2-Exo) that target fracture sites, accelerating fracture healing in mice (233). The inability of most drugs to cross the BBB limits therapeutic applications for central nervous system diseases. Studies have shown that M-EVs cross the BBB and deliver therapeutic cargo to the brain as carriers of central nervous system drugs, such as the therapeutic protein brain-derived neurotrophic factor (BDNF) or baicalin (BA) (217, 234, 235). In a mouse model of Alzheimer’s disease (AD), encapsulation of silymarin (Slb) into M-EVs delivered more SIb to the brain and enhanced its brain-targeting ability, leading to better efficacy and alleviation of cognitive impairment (236). Li et al. showed that Edaravone (Edv)-loaded M-EVs improve the bioavailability of Edv and efficiently and specifically deliver Edv into the ischemic brain, thereby enhancing neuroprotection in a rat model of permanent middle cerebral artery occlusion (PMCAO) (237). Collectively, these findings suggest that M-EVs can serve as novel brain-targeted drug delivery systems.

## 3 Conclusion and perspectives

Macrophages are innate immune cells distributed throughout the body that contribute to homeostasis and disease. M-EVs have the characteristics of parental cells, which play a wide role in the aforementioned diseases, mainly by mediating inflammation and immune responses, thus providing new ideas for the treatment of many diseases. M-EVs induce a pro-inflammatory microenvironment that favors a Th1 immune response, thereby inducing strong antigen-specific immune responses and making the immune system sensitive to cancer vaccines, demonstrating the potential of M-EVs as immunopotentiators for cancer vaccines (238). M-EVs have advantages, such as excellent stability, biosafety, compatibility, precise targeting, low immunogenicity, and the ability to cross the blood-brain barrier, making them highly effective drug carrier systems. This method of drug delivery selectively reduces non-specific uptake of the drug by normal cells, thereby reducing the risk of toxicity and adverse effects. The M-EVs derived from different types of macrophages have a significant ability to inhibit or promote inflammation, which will help to achieve a personalized and effective anti-cancer response.

However, there are still some challenges in their application, such as the low purity and yield of EVs, and further studies are needed to confirm their stability and safety. Notably, the internal environment of the body is very complex, and macrophages of different phenotypes exist simultaneously under different physiological and pathological conditions and jointly maintain physiological homeostasis. This suggests that M-EVs with different phenotypes coexist and work together.

Simultaneously, EVs contain a variety of bioactive substances including various proteins, lipids, and other biomolecules such as cytokines. These elements have the potential to influence the physiological state of recipient cells, thereby modulating their response to miRNAs. For instance, cytokines may alter miRNA expression patterns within recipient cells by activating distinct signal transduction pathways, consequently impacting the functional efficacy of miRNAs. However, most studies have focused on only one inclusion in a single phenotypic M-EV, such as only one or more miRNAs, or only one protein, mRNA, or lncRNA. A previous study found that the same miRNA from the same source of M-EVs can play a role in different diseases through different pathways, and different miRNAs can exert similar physiological effects by targeting the same target gene to regulate the same signaling pathway. Therefore, further research is required to confirm the use of a single miRNA as a potential biomarker for specific diseases. In forthcoming experimental designs, it may prove essential to rigorously regulate cytokine levels and cell state variables to facilitate an independent assessment of miRNA function. Employing high-throughput sequencing technologies could enable a comprehensive analysis of the phenotypic alterations in miRNAs and their target genes within recipient cells. This approach would be instrumental in elucidating the intricate interactions among various factors, thereby advancing our understanding of their collective impact. Further investigation into the transport mechanisms of miRNAs—encompassing their loading into and release from extracellular vesicles, as well as their interplay with cytokines—will significantly enhance our comprehension of the role that extracellular vesicles from macrophages play in disease pathogenesis. Such studies promise to elucidate the complex molecular dialogues that govern cellular communication and disease progression.
