# Supplementary figures and images for: Macrophage-derived extracellular vesicles as new players in chronic non-communicable diseases
Source: Front Immunol. 2025 Jan 17;15:1479330. doi: 10.3389/fimmu.2024.1479330 (PMC11782043; doi:10.3389/fimmu.2024.1479330)

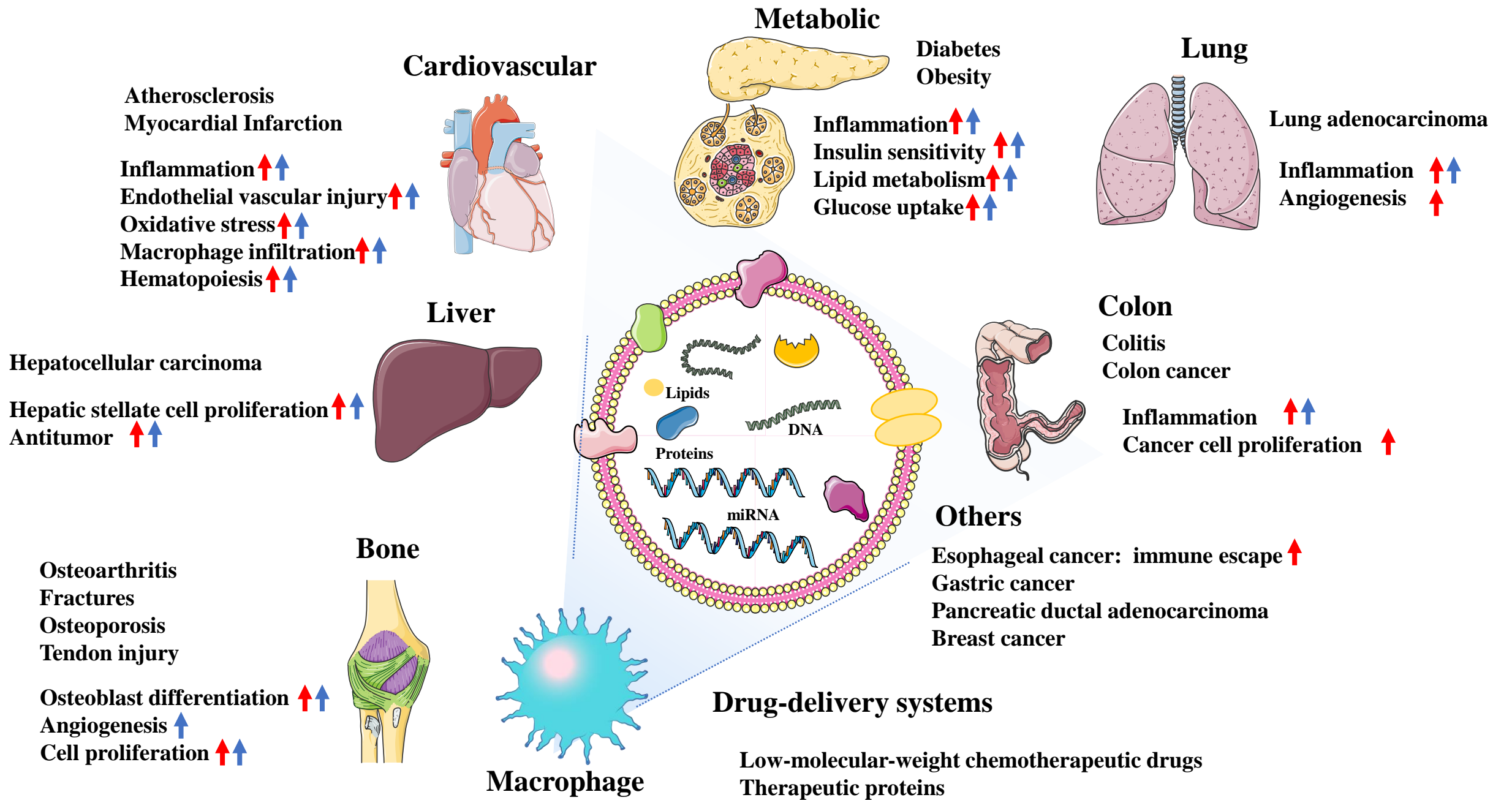

Supplement: Supplementary file 1 [file DataSheet1.pdf]
